# Cuprotosis-related signature predicts overall survival in clear cell renal cell carcinoma

**DOI:** 10.3389/fcell.2022.922995

**Published:** 2022-09-30

**Authors:** Fan Zhang, Junyu Lin, Dechao Feng, Jiayu Liang, Yiping Lu, Zhihong Liu, Xianding Wang

**Affiliations:** ^1^ Department of Urology, Institute of Urology, West China Hospital, Sichuan University, Chengdu, China; ^2^ West China Clinical Medical College, West China Hospital, Sichuan University, Chengdu, China

**Keywords:** cuprotosis, ccRCC, signature, cell death, prognosis

## Abstract

**Background:** Cuprotosis is a new form of programmed cell death induced by copper. We explored the correlation of cuprotosis with clear cell renal cell carcinoma (ccRCC) and constructed a cuprotosis-related signature to predict the prognosis of patients with ccRCC.

**Methods:** The clinical and transcriptomic data of ccRCC patients were downloaded from The Cancer Genome Atlas (TCGA), cBioPortal, and GEO databases, and cuprotosis-related gene sets were contained in the previous study. A cuprotosis-related signature was developed based on data from TCGA and verified by data from cBioPortal and GEO databases. The immune cell infiltrates and the corresponding signature risk scores were investigated. Two independent cohorts of clinical trials were analyzed to explore the correlation of the signature risk score with immune therapy response.

**Results:** A signature containing six cuprotosis-related genes was identified and can accurately predict the prognosis of ccRCC patients. Patients with downregulated copper-induced programmed death had a worse overall survival (hazard ratio: 1.90, 95% CI: 1.39–2.59, *p* < 0.001). The higher signature risk score was significantly associated with male gender (*p* = 0.026), higher tumor stage (*p* < 0.001), and higher histological grade (*p* < 0.001). Furthermore, the signature risk score was positively correlated with the infiltration of B cells, CD8^+^ T cells, NK cells, Tregs, and T cells, whereas it was negatively correlated with eosinophils, mast cells, and neutrophils. However, no correlation between cuprotosis and response to anti-PD-1 therapy was found.

**Conclusion:** We established a cuprotosis signature, which can predict the prognosis of patients with ccRCC. Cuprotosis was significantly correlated with immune cell infiltrates in ccRCC.

## Introduction

A new form of programmed cell death, namely, cuprotosis, has been established recently. Totally different from previously identified cell death mechanisms such as apoptosis or ferroptosis, cuprotosis is a copper-induced pathway of cell death which is dependent on mitochondrial respiration. The researchers found that cells that are more reliant on mitochondrial respiration are about 1,000-fold more sensitive to copper ionophores than cells undergoing glycolysis, indicating that cells undergoing glycolysis are less likely to suffer from cuprotosis (Tsvetkov et al., 2022). The Warburg effect, also known as the enhanced glycolysis, is a well-described metabolic reprogramming phenomenon in cancers (Weinhouse, 1956). Therefore, cancer cells are supposed to have an inactive cuprotosis, which might be an essential mechanism in the development of cancers. However, this theory has not been validated in specific cancers yet.

Renal cell carcinoma (RCC) refers to cancer that originates from the renal epithelium (Jonasch et al., 2014). It is one of the most common cancers worldwide, which accounts for more than 140,000 cancer-related deaths per year (Fitzmaurice et al., 2013). RCC encompasses more than 10 subtypes, among which clear cell renal cell carcinoma (ccRCC) is the most common subtype and accounts for the majority of the kidney cancer-related deaths (Hsieh et al., 2017). Metabolic reprogramming is well described in ccRCC, among which the Warburg effect is a hallmark of ccRCC (Wettersten et al., 2017). Therefore, cuprotosis might have played an important role in the development of ccRCC. A cuprotosis-associated signature has been already established in some cancers, such as head and neck squamous cell carcinoma (Yang et al., 2022) and soft tissue sarcoma (Han et al., 2022). Exploring the cuprotosis-related gene set systematically in ccRCC is of great importance for revealing the involvement of cuprotosis in the development of ccRCC and for potential copper ionophore therapy for cancers.

In the current study, we aimed to conduct a systematic and comprehensive research on the characteristics of the cuprotosis-related gene set in ccRCC to explore the role of copper-induced cell death in the development and prognosis of ccRCC and to establish a reliable prognostic model to predict the clinical outcomes of ccRCC.

## Materials and methods

### Dataset collection

Clinicopathological and sequencing data of 530 patients with ccRCC were contained from The Cancer Genome Atlas (TCGA) data portal (https://portal.gdc.cancer.gov/). Meanwhile, we retrieved a validation cohort (*n* = 446) from the cBioPortal online database (http://www.cbioportal.org/) and validated the findings (Gao et al., 2013). Moreover, the sequencing data and clinical information of two GEO datasets (GSE53757 and GSE150404) were also downloaded from the GEO database; the former consisted of 72 pairs of adjacent normal kidney tissue and ccRCC tissue, and the latter included 60 expression data of ccRCC samples at different tumor stages. A cuprotosis-related gene set was retrieved from the previous study involving ten genes: FDX1, LIAS, LIPT1, DLD, DLAT, PDHA1, PDHB, MTF1, GLS, and CDKN2A (Tsvetkov et al., 2022).

### Gene expression difference and signature identification

According to the data of 530 patients with ccRCC, the least absolute shrinkage and selection operator (LASSO) Cox regression algorithm was performed to construct a cuprotosis-related signature through the “glmnet” R package. We then verified the prognostic value of the signature by univariate and multivariate COX proportional hazards regression analysis involving clinical parameters. By using the “rms” and “survival” R packages, a nomogram and the calibration curves were constructed. Furthermore, we verified the signature using data from cBioPortal datasets (Cancer Genome Atlas Research Network, 2013). We utilized the webserver GEPIA to investigate the prognostic value of the cuprotosis-related genes (Tang et al., 2017). The Human Protein Atlas (HPA) was applied to retrieve immunohistochemistry to investigate the protein level of the genes involved in the signature.

### Differentially expressed gene identification and enrichment analysis

According to the two groups characterized by a cuprotosis-related signature, we identified the differentially expressed genes (DEGs) between those two groups and presented them in the form of volcano plots by the “ggplot” R package. After setting fold-change and adjusted *p* value at 1 and 0.05, respectively, we used the “ClusterProfiler” R package to conduct enrichment analyses (GO and KEGG analysis) to explore potential correlated enrichment terms. The Gene Set Enrichment Analysis (GSEA) was also conducted to explore mitochondrial metabolism and glycolysis enriched terms between groups with different risk scores.

### Cuprotosis signature and response to anti-PD-1 therapy

According to the strong correlation of cuprotosis with the immune microenvironment, we then explored the risk score of cuprotosis signature and response to anti-PD-1 therapy. We obtained RNA-seq and survival data from previous clinical trials, including NCT01668784 (a phase III clinical study comparing everolimus vs. nivolumab in previously treated metastatic ccRCC cases, CheckMate025) and NCT01354431 (a phase II study of nivolumab in metastatic ccRCC cases, CheckMate 010) (Braun et al., 2020). Based on tumor change after receiving nivolumab, patients were divided into three groups, including no clinical benefit (NCB), intermediate clinical benefit (ICB), and clinical benefit (CB) groups. ICB cases and CB cases were integrated and regarded as CB for the following investigation. The CB/NCB proportion between high- and low-risk score groups was analyzed by the chi-squared test, and the survival curves of patients who underwent anti-PD-1 therapy (nivolumab) were plotted.

## Results

### Data collection and cuprotosis-related signature construction

To explore the role of cuprotosis in ccRCC, we obtained a cohort of 530 patients using RNA sequencing data and clinicopathological information from TCGA database. A cuprotosis-related gene set involving 10 genes was then obtained.

**FIGURE 1:**
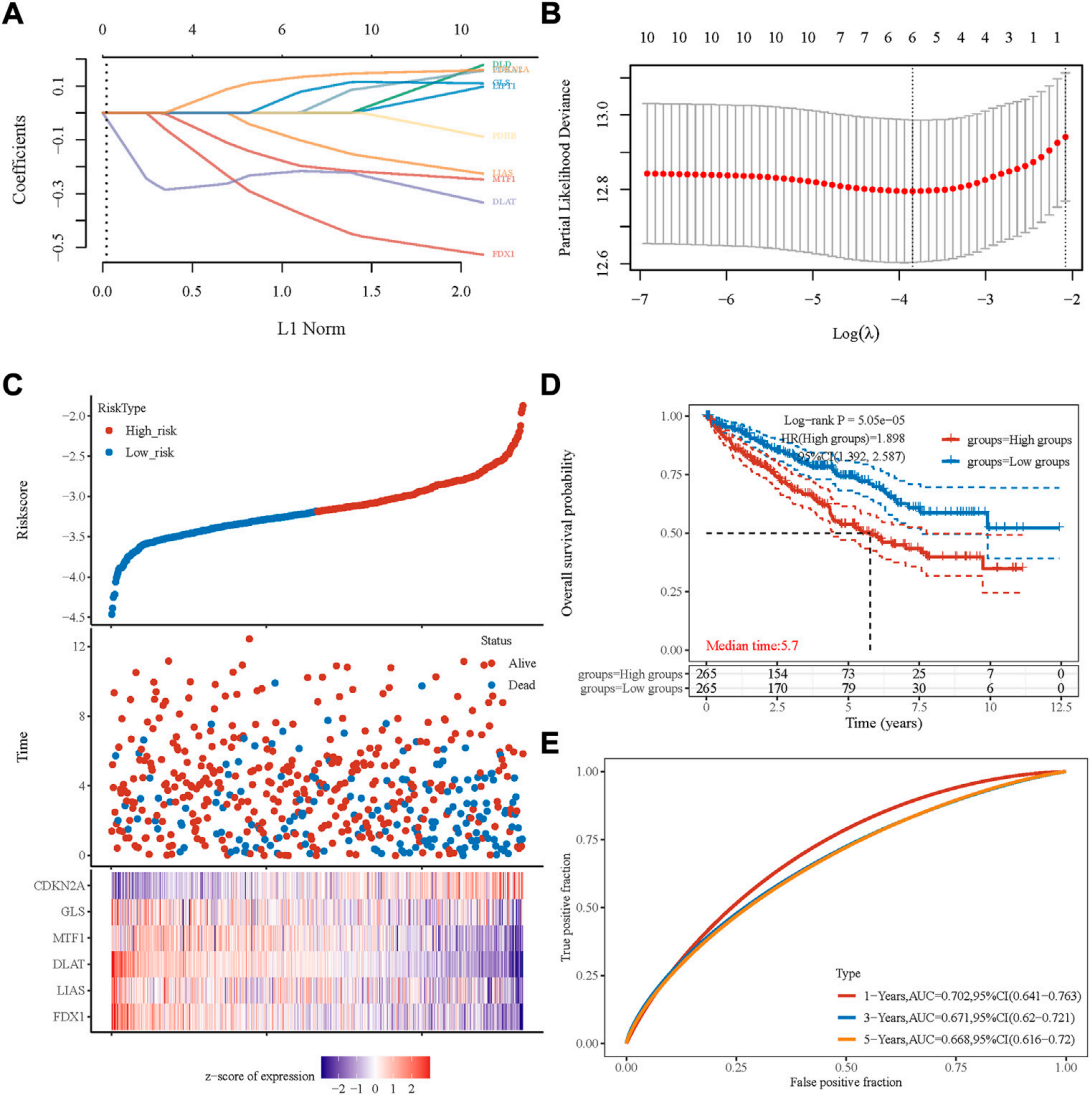
Prognostic signature was established based on six prognostic cuprotosis-related genes. **(A)** LASSO coefficient profiles of the genes associated with the cuprotosis of ccRCC. **(B)** Partial likelihood deviance is plotted versus log (*λ*). **(C)** The risk score of each sample is based on the cuprotosis-related signature. Patients were divided into low-risk and high-risk groups according to the median value of the risk score. High/low expression levels of six genes, which were involved in the prognostic signature, are shown in red/blue in each sample. **(D)** Kaplan–Meier curve of overall survival differences stratified by the signature risk score. **(E)** ROC curves of the signature for overall survival rates at 1, 3, and 5 years. LASSO, least absolute shrinkage, and selection operator; ROC, receiver operating characteristic.

A cuprotosis-related gene signature was constructed for prognosis prediction. Based on ten cuprotosis-related genes, LASSO analysis was conducted (Figures 1A,B), and we obtained six genes associated with the prognosis of patients with ccRCC. The cuprotosis-related gene-based prognostic signature was calculated as follows: risk score = (−0.3233 × FDX1 expression) + (−0.0652× LIAS expression) + (−0.2261× DLAT expression) + (−0.1638× MTF1 expression) + (0.0304× GLS expression) + (0.1192× CDKN2A expression).

Based on the median value of the risk score, patients with ccRCC could be categorized into low-risk and high-risk groups (Figure 1C). The Kaplan–Meier curve indicated that patients in the high-risk group had a significantly poorer OS rate than those in the low-risk group (Figure 1D, *p* < 0.001), and the AUCs for 1-, 3-, and 5-year OS rates were 0.702, 0.671, and 0.668, respectively (Figure 1E). The multivariate and univariate Cox regression analyses of the cuprotosis-related signature and other clinicopathological characteristics for OS are presented in Table 1. The signature risk score was an independent risk factor for the prognosis of patients with ccRCC (HR: 1.473, 95% CI: 1.069–2.028, *p* = 0.018).

**TABLE 1:** Multivariate and univariate Cox regression analyses of the signature risk score and other clinicopathologic characteristics for OS in the TCGA cohort.

Characteristic	Univariate analysis		Multivariate analysis	
	HR (95% CI)	*p* value	HR (95% CI)	*p* value
Age	1.028 (1.015–1.041)	<0.001	1.029 (1.015–1.043)	<0.001
Gender		0.828		
F	Reference			
M	0.966 (0.705–1.322)	0.828		
Race		0.804		
WHITE	Reference			
BLACK	0.923 (0.500–1.704)	0.798		
ASIAN	0.540 (0.075–3.865)	0.539		
pTNM stage		<0.001		
I–II	Reference			
III–IV	3.832 (2.781–5.281)	<0.001	3.020 (2.150–4.243)	<0.001
Grade		<0.001		
G3–G4	Reference			
G1–G2	0.367 (0.259–0.521)	<0.001	0.583 (0.403–0.843)	0.004
Risk score		<0.001		
Low risk	Reference			
High risk	1.874 (1.369–2.565)	<0.001	1.473 (1.069–2.028)	0.018

### Nomogram construction

**FIGURE 2:**
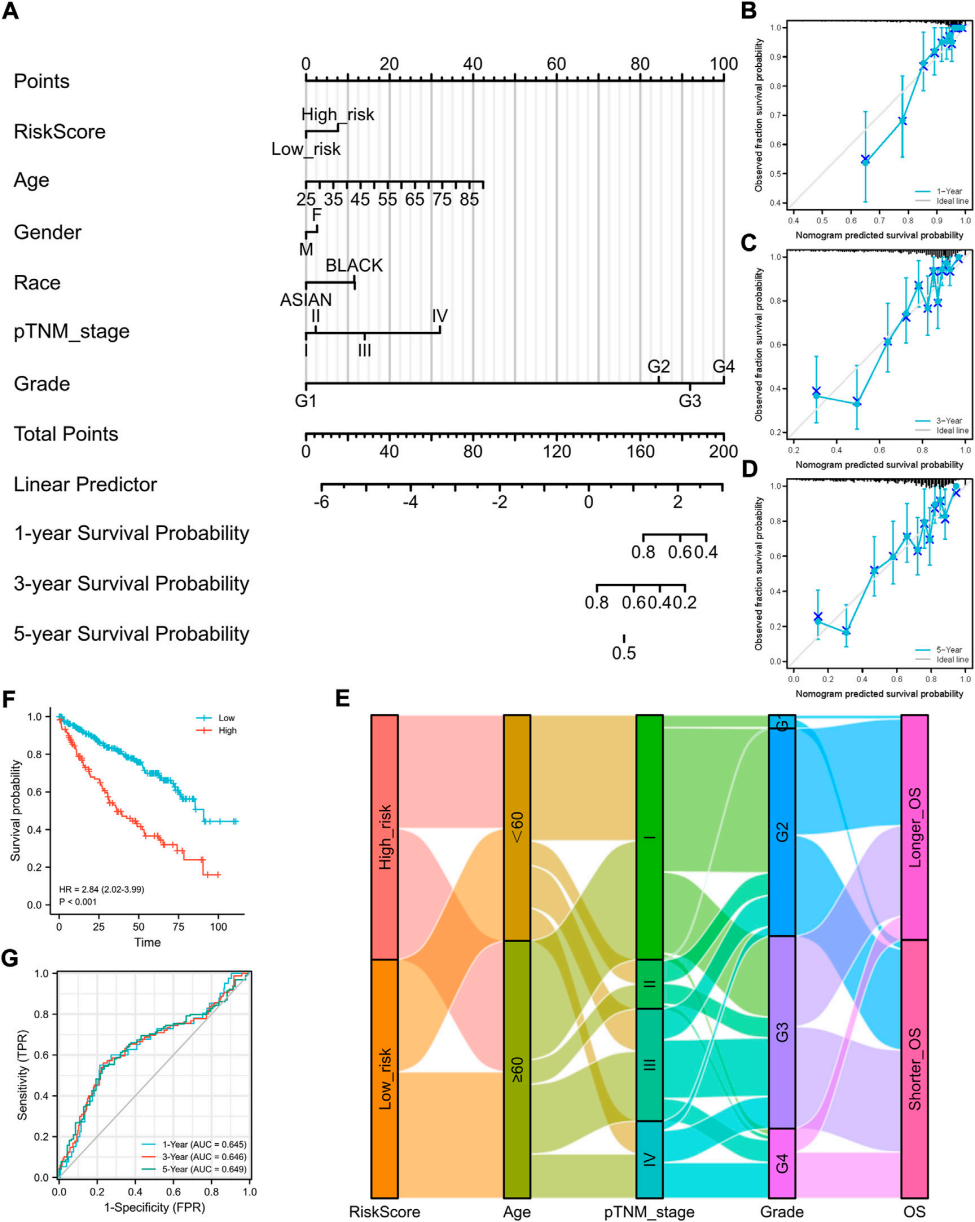
Construction of a nomogram and the independent signature validation. **(A)** Nomogram for predicting 1-, 3-, or 5-year OS rates in patients with ccRCC. **(B)** Calibration plots for predicting 1-year OS rate. **(C)** Calibration plots for predicting 3-year OS rate. **(D)** Calibration plots for predicting 5-year OS rate. **(E)** Sankey Diagram showing the association between signature risk scores and clinicopathological characteristics. **(F)** Validation of the signature in overall survival based on data from the cBioPortal online database. **(G)** ROC curves of the signature validation for overall survival rates at 1, 3, and 5 years. OS, overall survival; ROC, receiver operating characteristic.

To better predict the prognostic value of the signature in patients with ccRCC, a nomogram using available clinicopathological parameters and the risk score of the signature was constructed (Figure 2A). Moreover, calibration curves using 1-, 3-, and 5-year survival rates were developed to estimate the accuracy of the nomogram (Figures 2B–D). The association between signature risk scores and clinicopathological characteristics was presented in the form of a Sankey diagram (Figure 2E). A higher signature risk score was significantly correlated with male gender (*p* = 0.026), higher tumor stage (*p* < 0.001), and higher histological grade (*p* < 0.001, Supplementary Material S1).

### Enrichment analysis

**FIGURE 3:**
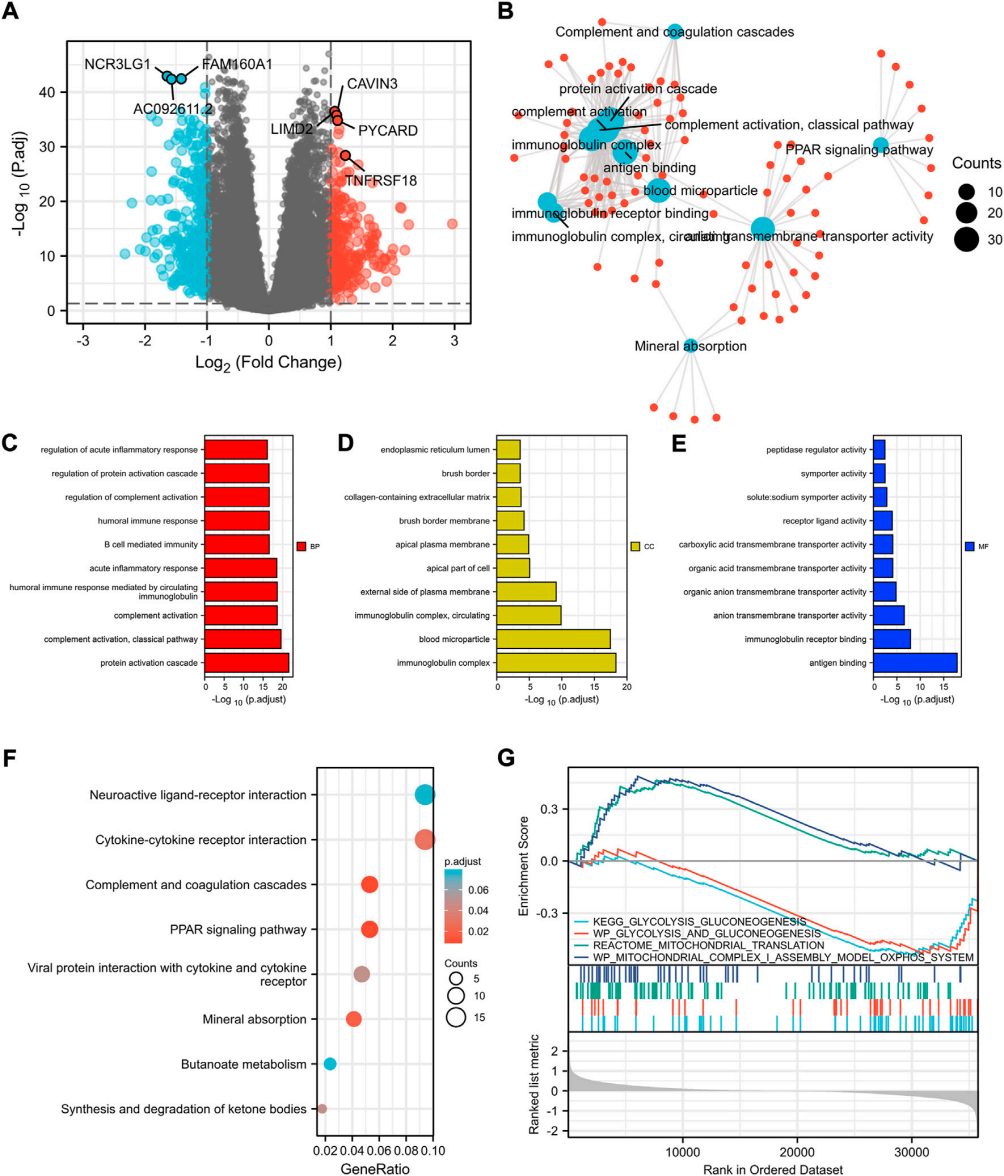
Identification of differentially expressed genes between the high- and low-risk patients according to cuprotosis-related signature and functional enrichment analysis. **(A)** Volcano plot of DEGs between high- and low-risk patients with ccRCC. Red and blue points represent up- and downregulated genes with statistical significance, respectively. **(B)** Network for comparing biological themes among gene clusters. GO analysis of DEGs between high- and low-risk patients including biological process **(C)**, cellular components **(D)**, molecular function **(E)**, and KEGG analysis **(F)**. **(G)** GSEA results of mitochondrial metabolism and glycolysis terms. DEGs, differentially expressed genes; GSEA, Gene Set Enrichment Analysis.

To explore the underlying mechanisms of the differences between high- and low-risk patients, DEGs between the two groups were identified. As shown in Figure 3A, the volcano plot indicated the upregulated genes (CAVIN3, LIMD2, PYCARD, etc.) and downregulated genes (NCR3LG1, FAM160A1, etc.) in the high-risk group compared to the low-risk group.

Moreover, with the thresholds of the fold-change value and adjusted *p* value set at 1 and 0.05, up- and downregulated genes were selected for functional enrichment analysis (Figure 3B). The results of GO analysis indicated that the most enriched terms in biological process (BP, Figure 3C), molecular function (MF, Figure 3D), and cellular component (CC, Figure 3E) were strongly correlated with immune terms, mainly enriched in protein activation cascade, complement activation, immunoglobulin complex, and antigen binding. KEGG analysis of the most relevant signaling pathways was mainly associated with the PPAR signaling pathway and complement and coagulation cascades (Figure 3F). The GSEA indicated mitochondrial metabolism was activated in the low-risk group and glycolysis was inhibited (Figure 3G), which was consistent with the previous finding that cuprotosis was more activated in cells relying on mitochondrial respiration than those relying on glycolysis.

### Correlation of the signature risk score with immune cell infiltrates and response to anti-PD-1 immunotherapy

**FIGURE 4:**
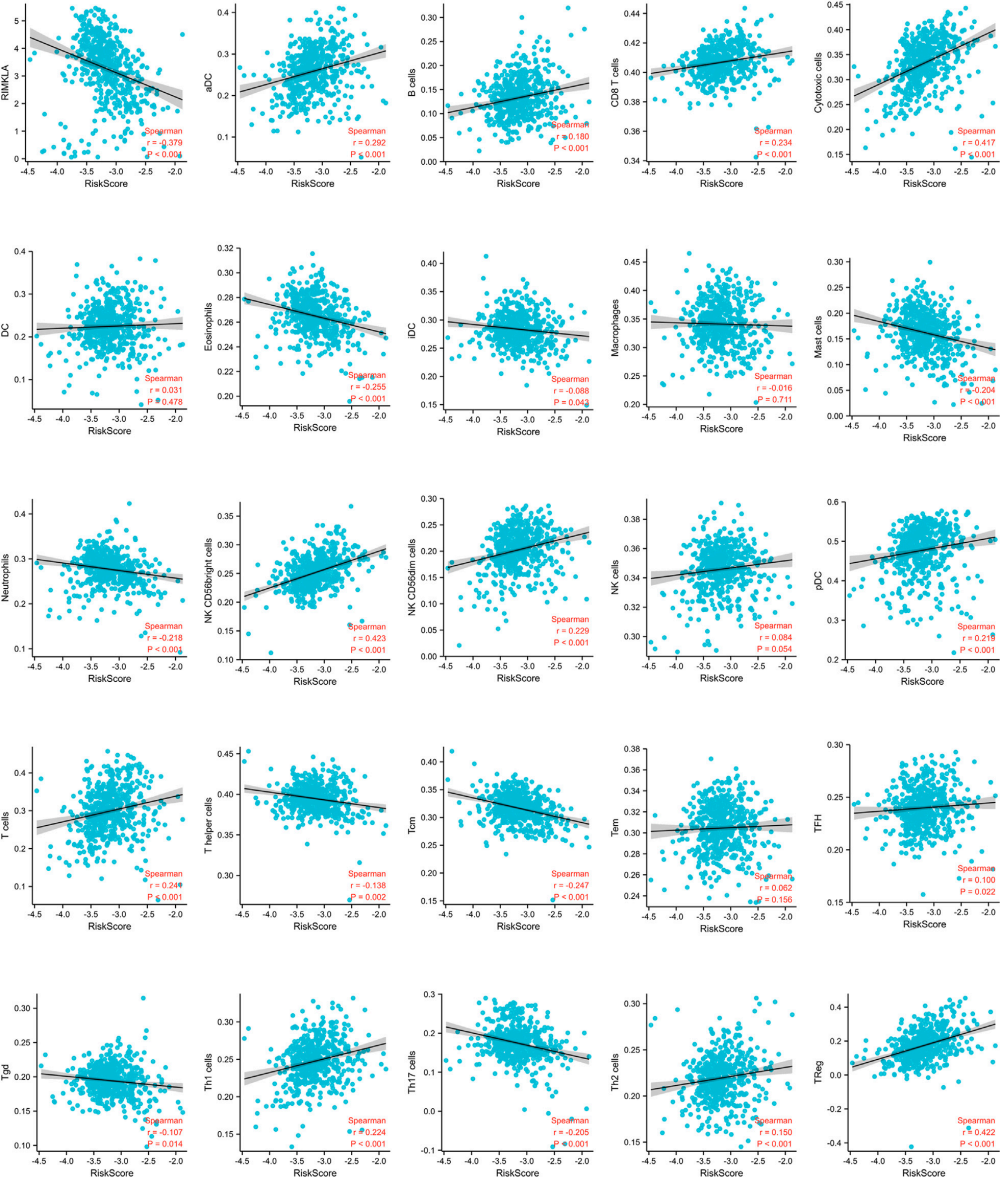
Correlation of immune cell infiltrates with the signature risk score.

We found a strong correlation between the signature risk score and the immune microenvironment according to TCGA datasets (Figure 4). The signature risk score was positively correlated with the infiltration of B cells, CD8^+^ T cells, NK cells, T.cells, and Tregs, whereas it was negatively correlated with eosinophils, mast cells, and neutrophils. However, based on the sequencing and prognostic data of 156 advanced ccRCC cases treated with nivolumab, we did not observe a correlation between the signature and response to anti-PD-1 therapy. No association of the risk score with OS or PFS in patients who received anti-PD-1 therapy was found (Supplementary Material S2).

### Expression differences and survival curves

**FIGURE 5:**
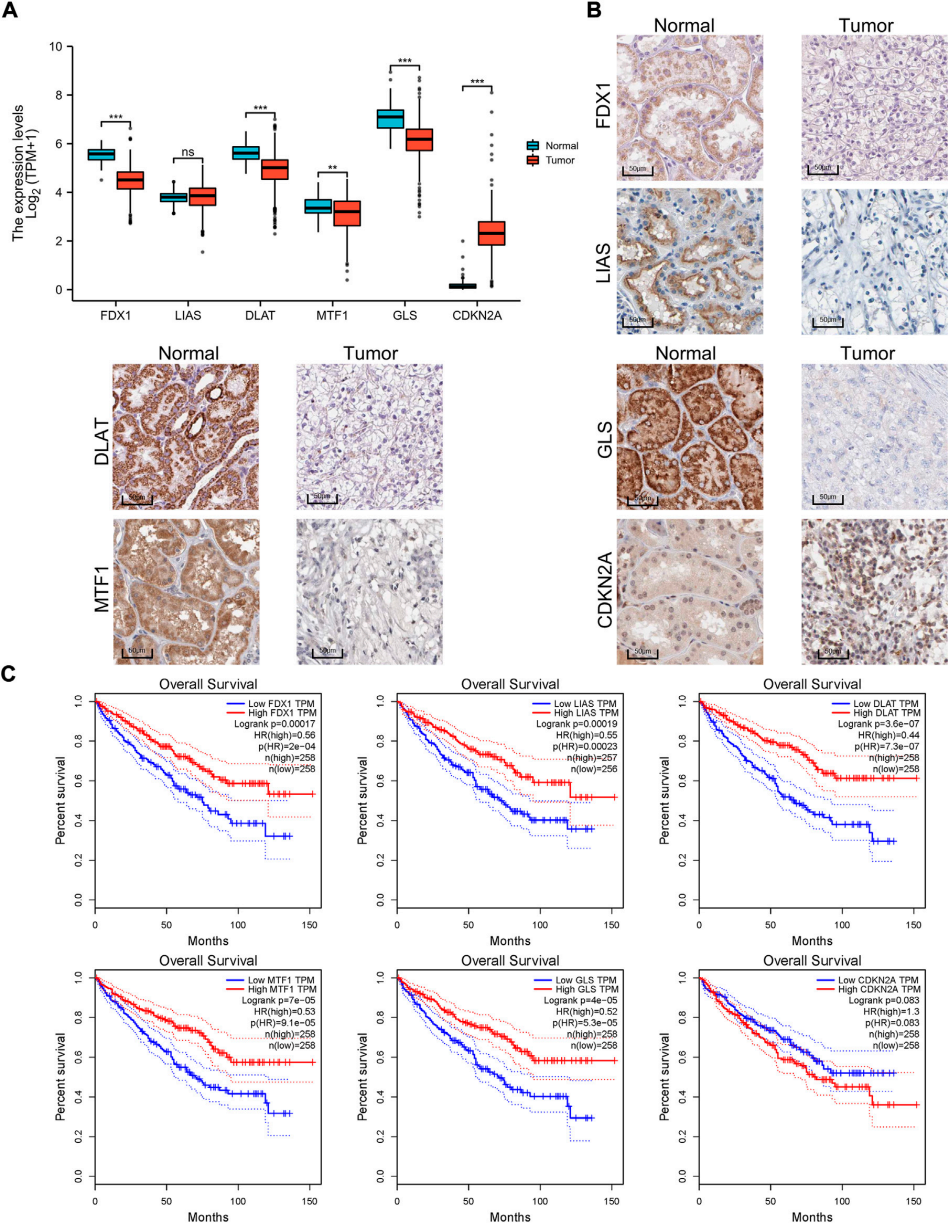
Expression differences and survival of the signature genes. **(A)** Box plot of the expression difference of signature genes between normal and ccRCC tissue according to TCGA sequencing data. **(B)** IHC of FDX1, LIAS, DLAT, MTF1, GLS, and CDKN2A between tumor and normal tissue according to the Human Protein Atlas cohort. **(C)** Survival curves of six genes involved in the prognostic signature. IHC, immunohistochemistry.

Furthermore, the different expression patterns between the normal and tumor tissues of six genes involved in the signature were explored at the mRNA (Figure 5A) and protein level (Figure 5B). Of the six cuprotosis-related genes, we found that *FDX1*, *LIAS*, *DLAT*, *MTF1*, and *GLS* were differentially expressed between tumor and normal tissue and were upregulated in normal tissue. This result may indicate that copper-induced cell death was inhibited in ccRCC cells compared with normal tissue. A survival analysis of the six genes for OS was also performed (Figure 5C). High FDX1, LIAS, DLAT, MTF1, and GLS levels were significantly positively correlated with a better OS rate, whereas high expression levels of CDKN2A served as a prognostic marker correlated with a worse OS rate.

### Signature validation

To ensure the prediction value of the signature, an independent cohort from the cBioPortal online database served as a validation set to verify our results. Survival curves showed similar results, and significantly worse OS was observed in patients from the high-risk group than those from the low-risk group (Figure 2F). The AUCs for 1-, 3-, and 5-year OS rates in the validation cohort were 0.645, 0.646, and 0.639, respectively (Figure 2G). We also validated our established prognostic signature in patients with stage III–IV ccRCC (*n* = 202). We found that this prognostic signature is still applicable in patients with advanced ccRCC; that is, patients with higher risk scores have a significantly poorer prognosis (Supplementary Material S3).

Furthermore, we have validated the results using another two Gene Expression Omnibus (GEO) datasets of ccRCC, namely, GSE53757 and GSE150404. The GSE53757 dataset consisted of 72 pairs of adjacent normal kidney tissues and ccRCC tissues. Using the GSE53757 dataset, we detected that the expression of CDKN2A was higher in ccRCC tissue compared to normal kidney tissue, and the expressions of FDX1, LIAS, DLAT, and GLS were lower in ccRCC tissue compared to normal kidney tissue (Supplementary Material S4), which was consistent with our original results. The GSE150404 dataset consisted of 60 ccRCC tissues of different tumor stages. Using the GSE150404 dataset, we used the signature we established in the current study to calculate the risk score for each patient, and we detected that the risk score was significantly associated with the pathological stage (Supplementary Material S5), which indicated that the high-risk group had a poorer prognosis than the low-risk group. Unfortunately, the clinical information of samples of this dataset contained only tumor stage, and no survival data of patients were available.

## Discussion

Cuprotosis is a newly detected mechanism of cell death induced by copper. Excess copper can bind directly to lipoylated components of the tricarboxylic acid (TCA) cycle, leading to mitochondrial protein aggregation and triggering the pathway of cell death (Tsvetkov et al., 2022). Therefore, cuprotosis highly depends on mitochondrial respiration. The Warburg effect is one of the most well described characteristics of ccRCC, indicating that cells in ccRCC rely more on glycolysis rather than mitochondrial respiration (Wettersten et al., 2017). In the current study, using the RNA sequencing data from TCGA and cBioPortal online databases, a cuprotosis-related signature was established to stratify patients into high- or low-risk of poor prognosis. With the application of a combination of lasso regression, a signature of six genes showed a powerful effect on survival prediction. Patients in the high-risk group had a significantly worse OS rate compared to those in the low-risk group. Thus, a significant correlation was established between the expression levels of the cuprotosis-related genes and the prognosis of patients with ccRCC, which has confirmed the role of cuprotosis in ccRCC.

Of the six cuprotosis-related genes included in the signature, we found *FDX1*, *LIAS*, *DLAT*, *MTF1*, and *GLS* to be differentially expressed between tumor and normal tissues at the protein level. *FDX1* encodes ferredoxin 1 (FDX1), a member of the mammalian adrenodoxin. FDX1 is associated with mitochondrial cytochrome and has important roles in steroidogenesis and Fe–S cluster biosynthesis (Sheftel et al., 2010). Acting as a reductase, it can reduce Cu^2+^ to Cu^1+^, the more toxic form. FDX1 has also proved to be an upstream regulator of protein lipoylation (Tsvetkov et al., 2022). A study found that FDX1 can impact the prognosis of lung adenocarcinoma (Zhang et al., 2021). *LIAS* encodes lipoyl synthase, an Fe–S cluster protein and a member of the radical S-adenosylmethionine (SAM) superfamily, which is a vital component of the lipoic acid pathway (Hendricks et al., 2021). *DLAT* encodes dihydrolipoamide S-acetyltransferase (DLAT), which is a subunit of the pyruvate dehydrogenase (PDH) complex and a protein target of lipoylation. Previous studies revealed that DLAT is reprogrammed in cancer and plays a role in cancer cell proliferation (Goh et al., 2015). Cuprotosis depends highly on the protein lipoylation machinery (Tsvetkov et al., 2022). The different expression of the three lipoylation-related proteins between tumor and normal tissues detected in the current study indicated that ccRCC has low levels of lipoylated proteins, leading to decreased cuprotosis. *MTF1* encodes metal-regulatory transcription factor 1 (MTF1). A study found that MTF1 was upregulated in ovarian cancer, and its high expression was associated with poor patient survival and disease relapse (Ji et al., 2018). *GLS* encodes glutaminase (GLS), a crucial enzyme in the regulation of glutamine metabolism. GLS has been reported to have a crucial impact on cancer development (Zhang et al., 2019). In addition, survival analysis showed that the high expression level of *CDKN2A* was correlated with a worse OS rate in ccRCC. CDKN2A is involved in cell-cycle progression and has been validated to play an important role in many kinds of cancers, including pancreatic cancer (Gu et al., 2020), sebaceous gland carcinoma of the eyelid (Yunoki et al., 2020) and so on. Our results further confirmed the role of CDKN2A in ccRCC. However, further studies are needed to reveal the exact mechanisms of how these genes affect the development and progression of ccRCC.

In addition, GSEA showed that the mitochondrial metabolism was activated in the low-risk group and glycolysis was inhibited, which was consistent with the previous finding that cuprotosis was more activated in cells relying on mitochondrial respiration than those relying on glycolysis. Functional enrichment analysis also revealed a strong association between the expression levels of the cuprotosis-related genes and immune responses, suggesting an interface between cuprotosis and the tumor immune microenvironment. Further analysis revealed that the signature risk score was positively correlated with the infiltration of CD8^+^ T cells. Given that high levels of tumor CD8 T-cell infiltration have been detected to correlate with a worse prognosis in patients with ccRCC (Remark et al., 2013), this might be one of the rational explanation for the poor prognosis observed in patients with the high risk score. Further studies are needed to explore the exact mechanism of the link between immune responses and cuprotosis.

There are some limitations that should be acknowledged in the current study. First, a few cases with higher tumor grade and pathologic stages were involved in the clinical trial cohort, which may result in the limited prognostic value of the signature in these patients. Second, most analyses were performed at the mRNA level, and further analysis at the protein level was imperative. Third, our results were mainly performed based on TCGA and cBioPortal datasets. Although the large scale of cases from these databases may somehow decrease the risk of bias, another independent cohort is needed to validate our results and minimize the bias.

In conclusion, our study revealed that cuprotosis has a close correlation with the immune microenvironment and the prognosis of patients with ccRCC. The cuprotosis-related gene signature established in the current study can be used to predict the survival of patients with ccRCC.

## Data Availability

The original contributions presented in the study are included in the article/Supplementary Material; further inquiries can be directed to the corresponding authors.
