# Supplementary material for: Cuprotosis-related signature predicts overall survival in clear cell renal cell carcinoma
Source: Front Cell Dev Biol. 2022 Sep 30;10:922995. doi: 10.3389/fcell.2022.922995 (PMC9562982; doi:10.3389/fcell.2022.922995)
Supplement: Supplementary file 1 [file Table1.docx]

Supplementary material 1


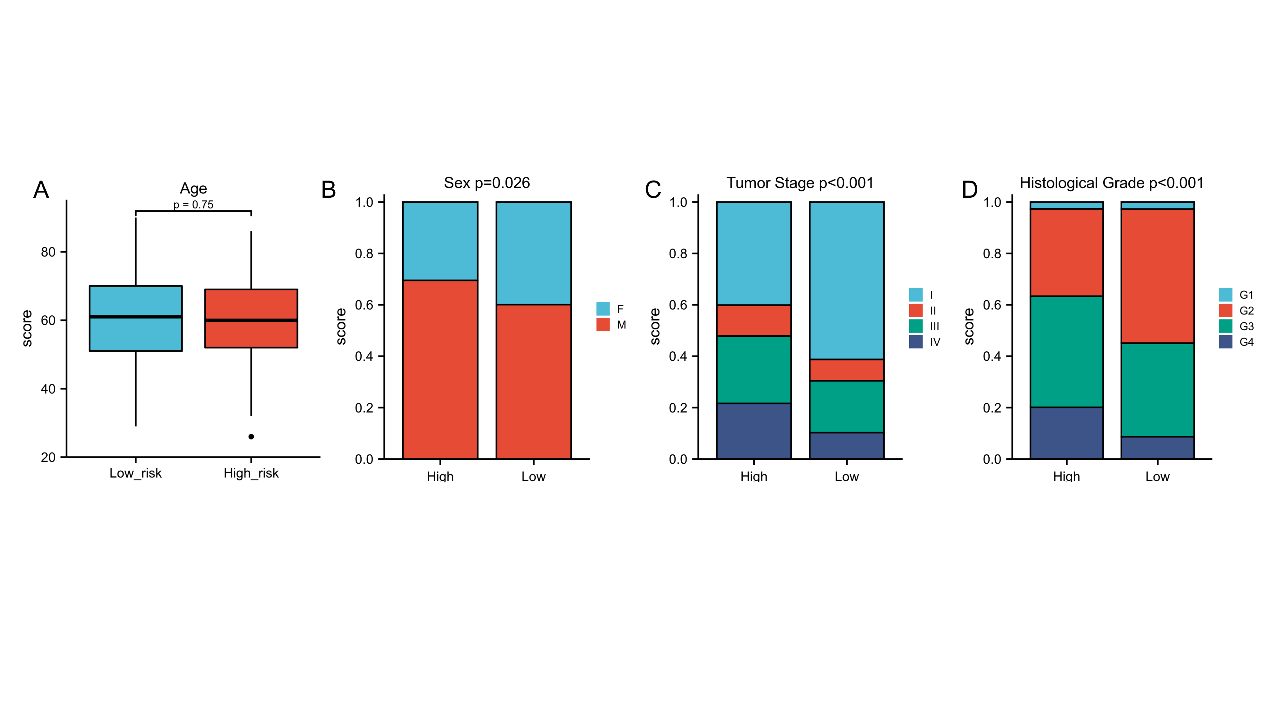


Supplementary material 1: Correlation of signature risk score with age (A), sex (B), tumor stage (C), and histological grade (D).

Supplementary material 2


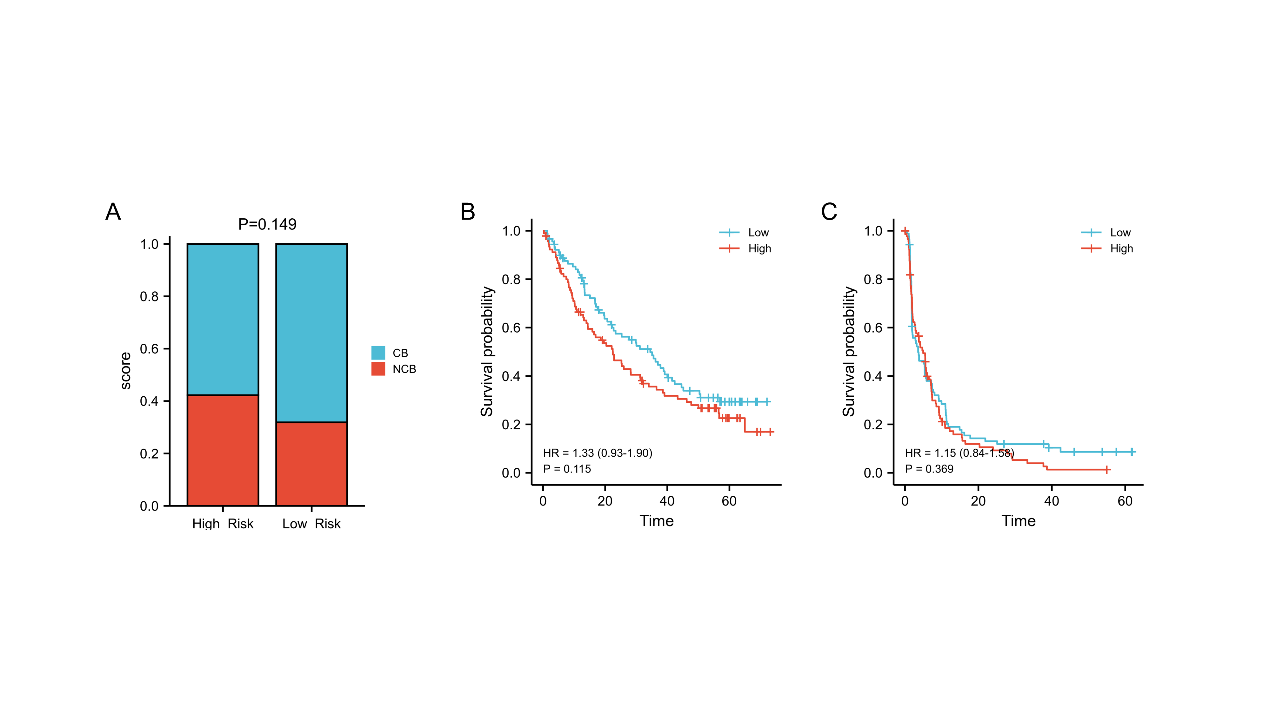


Supplementary material 2: Response to anti-PD-1 immunotherapy in high-risk patients with ccRCC. A: Proportion of CB versus NCB cases in patients with high or low risk scores. B: OS in high- and low-risk patients who underwent anti-PD-1 immunotherapy. C: PFS in high- and low-risk patients who underwent anti-PD-1 immunotherapy.

Supplementary material 3


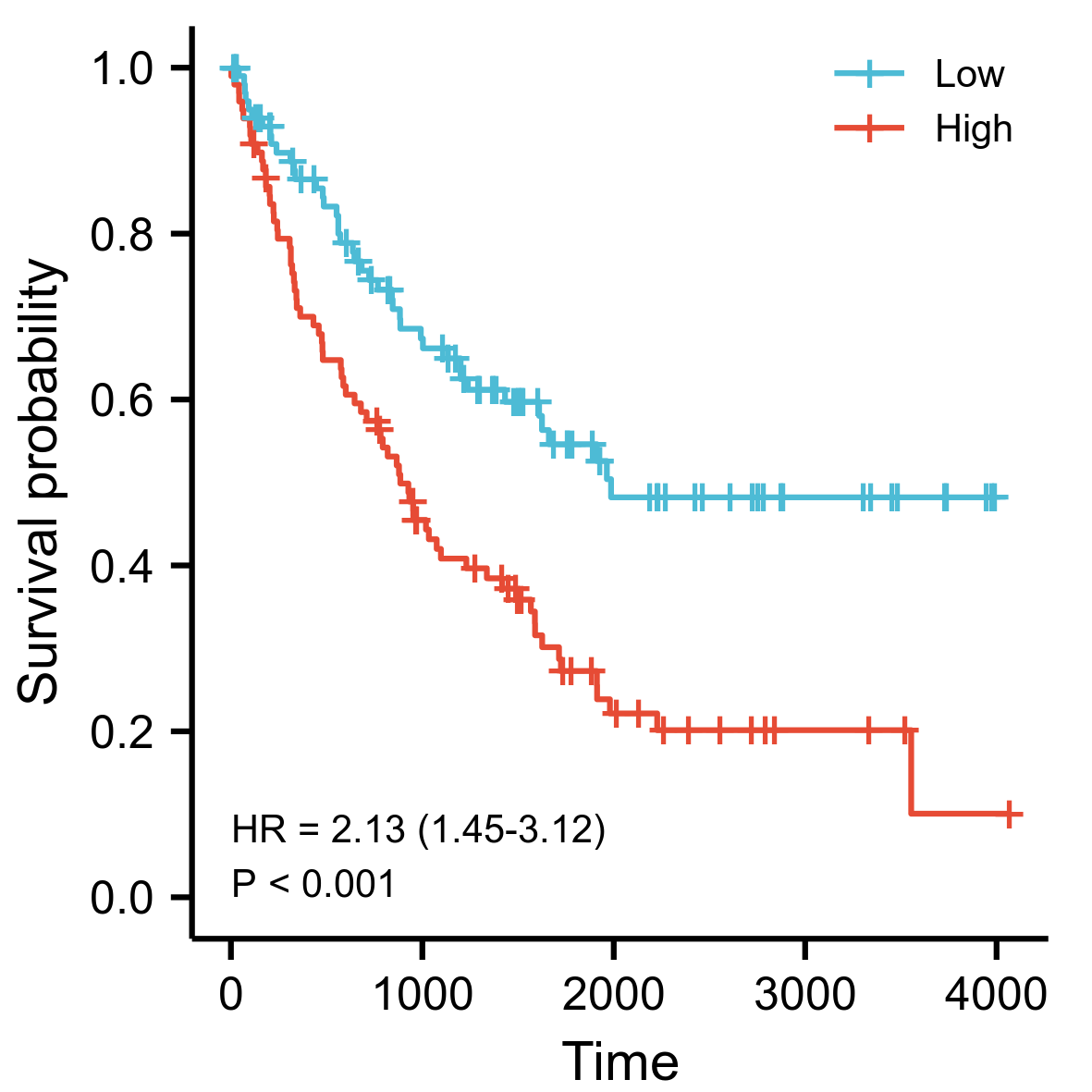


Supplementary material 3: Validation of the signature in overall survival based on data from patients with advanced ccRCC.

Supplementary material 4


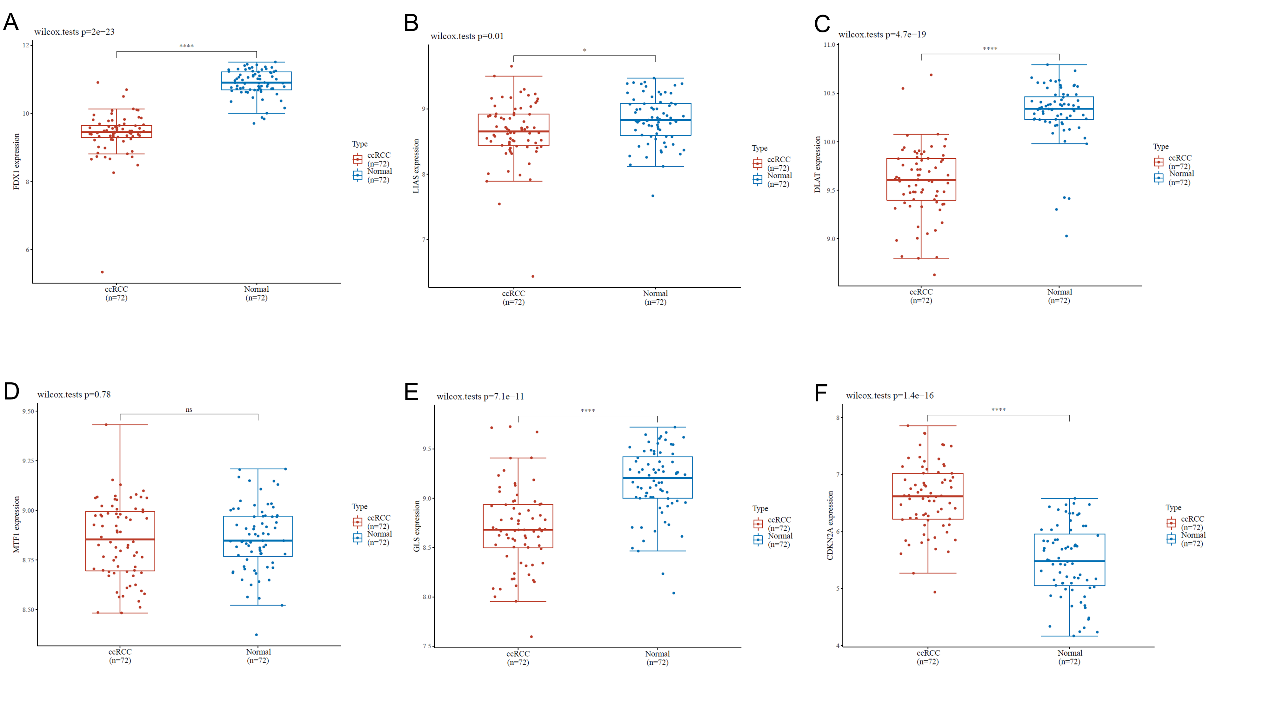


Supplementary material 4: Differential expression analysis and validation in GEO datasets. Boxplots of the expression of FDX1 (A), LIAS (B), DLAT (C), MTF1 (D), GLS (E), and CDKN2A (F) in GSE53757.

Supplementary material 5


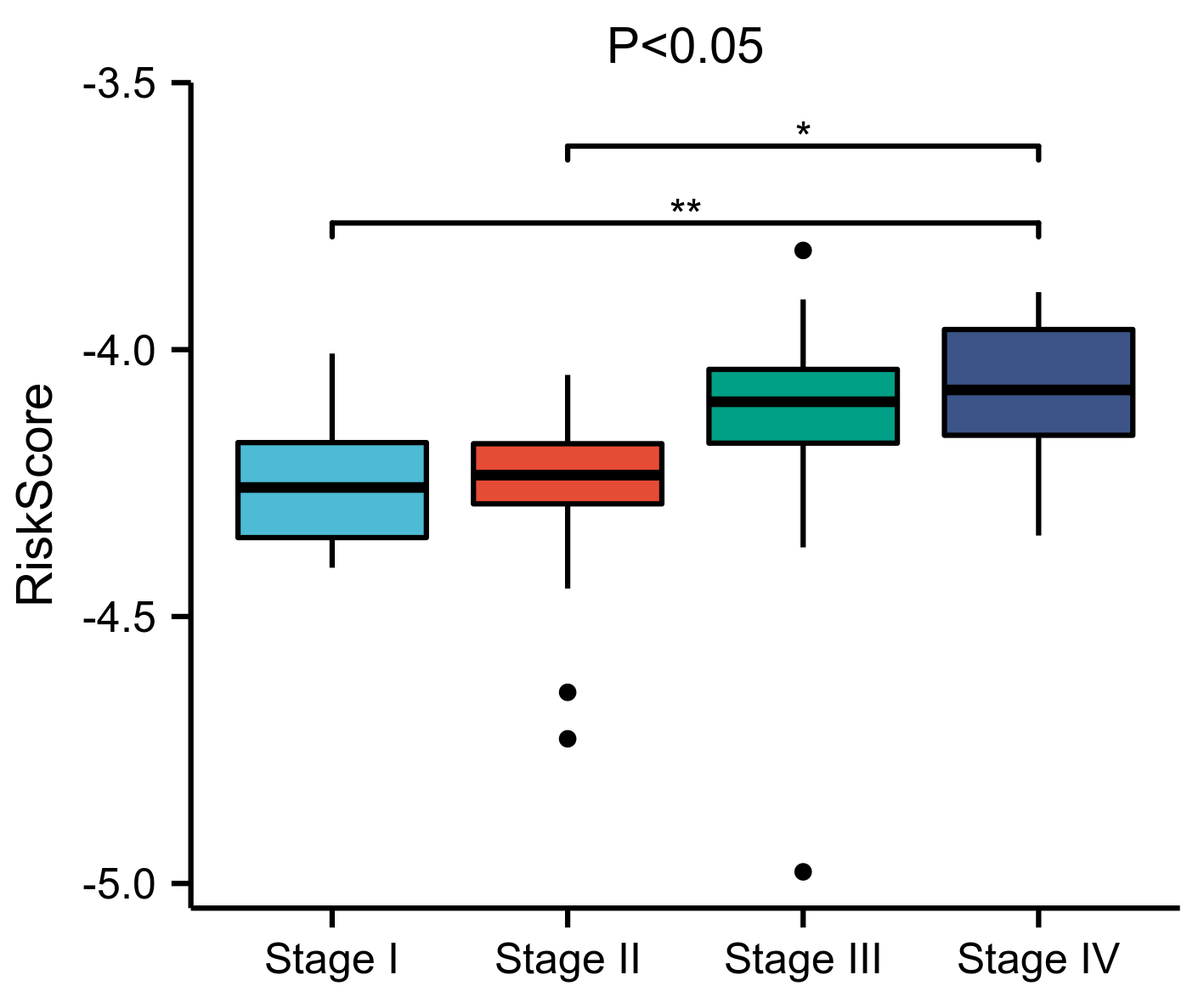


Supplementary material 5: Correlation between signature risk scores and ccRCC stages based on data from GSE150404.
