# Supplementary material for: Cuprotosis-related signature predicts overall survival in clear cell renal cell carcinoma
Source: Front Cell Dev Biol. 2022 Sep 30;10:922995. doi: 10.3389/fcell.2022.922995 (PMC9562982; doi:10.3389/fcell.2022.922995)
Supplement: Supplementary file 2 [file Table2.DOCX]

**Lasso regression**

# library(tidyverse)

library(survival)

library(glmnet)

library(readxl)

## read data

data <- read_xlsx("~/file.xlsx")

head(data)

# event time Gene 1 Gene 2 Gene 3 Gene 4 Gene 5 Gene 6 Gene 7 Gene 8 Gene 9 Gene 10 Gene 11

# 1 1 306 -0.02228062 -2.7704356 -0.4670543 0.6597100 -0.5151171 0.02662303 0.9648682 -1.0649103 0.1261321 -1.2472454 0.2857966

# 2 1 455 -1.18321709 -0.3161180 0.6060377 -0.3035985 0.1487279 -1.21013808 1.2133766 -0.1951160 0.6075327 0.3840250 0.6994784

# 3 0 1010 -0.62297439 1.8458862 1.5176689 -0.8392173 -0.2736995 -1.91479449 0.9869319 -0.1859204 0.2624222 0.8615537 -0.5557269

# 4 1 210 -0.96143221 -0.1361290 0.7070270 -2.2407777 -0.1158965 -1.67839318 0.5813433 1.2488396 -0.4216805 -0.3247411 -0.1496818

# 5 1 883 -2.00905791 0.7544725 -1.3601112 0.7434566 1.2420130 0.37301567 0.6557969 -0.6545812 -0.7919030 -0.7085475 -2.8072731

# 6 0 1022 0.79356585 -0.2366209 -0.5012338 0.9380560 -1.2196590 -1.62508198 0.3280815 1.0461292 -0.6751357 1.4510194 -0.7491115

set.seed(2021)

cvfit = cv.glmnet(x = as.matrix(data[,c(-1, -2)]),

y = Surv(time = data$time, event = data$event), family = "cox",

alpha = 1)

# cvfit

# Call: cv.glmnet(x = as.matrix(data[, c(-1, -2)]), y = Surv(time = data$time, event = data$event), family = "cox", alpha = 1)

#

# Measure: Partial Likelihood Deviance

#

# Lambda Index Measure SE Nonzero

# min 0.09095 7 10.16 0.1983 4

# 1se 0.15894 1 10.17 0.1893 0

## Check Coefficients

# coef(cvfit, s = "lambda.1se")

coef.min = coef(cvfit, s = "lambda.min")

## riskscore

as.matrix(dat1[,c(-1,-2)]) %*% as.matrix(coef.min)

## plot

plot(cvfit)

plot(cvfit$glmnet.fit, xvar = "norm")

plot(cvfit$glmnet.fit, xvar = "lambda")

# library(tidyverse)

library(survival)

library(glmnet)

library(readxl)

## read data

data <- read_xlsx("~/file.xlsx")

head(data)

# event time Gene 1 Gene 2 Gene 3 Gene 4 Gene 5 Gene 6 Gene 7 Gene 8 Gene 9 Gene 10 Gene 11

# 1 1 306 -0.02228062 -2.7704356 -0.4670543 0.6597100 -0.5151171 0.02662303 0.9648682 -1.0649103 0.1261321 -1.2472454 0.2857966

# 2 1 455 -1.18321709 -0.3161180 0.6060377 -0.3035985 0.1487279 -1.21013808 1.2133766 -0.1951160 0.6075327 0.3840250 0.6994784

# 3 0 1010 -0.62297439 1.8458862 1.5176689 -0.8392173 -0.2736995 -1.91479449 0.9869319 -0.1859204 0.2624222 0.8615537 -0.5557269

# 4 1 210 -0.96143221 -0.1361290 0.7070270 -2.2407777 -0.1158965 -1.67839318 0.5813433 1.2488396 -0.4216805 -0.3247411 -0.1496818

# 5 1 883 -2.00905791 0.7544725 -1.3601112 0.7434566 1.2420130 0.37301567 0.6557969 -0.6545812 -0.7919030 -0.7085475 -2.8072731

# 6 0 1022 0.79356585 -0.2366209 -0.5012338 0.9380560 -1.2196590 -1.62508198 0.3280815 1.0461292 -0.6751357 1.4510194 -0.7491115

set.seed(2021)

cvfit = cv.glmnet(x = as.matrix(data[,c(-1, -2)]),

y = Surv(time = data$time, event = data$event), family = "cox",

alpha = 1)

# cvfit

# Call: cv.glmnet(x = as.matrix(data[, c(-1, -2)]), y = Surv(time = data$time, event = data$event), family = "cox", alpha = 1)

#

# Measure: Partial Likelihood Deviance

#

# Lambda Index Measure SE Nonzero

# min 0.09095 7 10.16 0.1983 4

# 1se 0.15894 1 10.17 0.1893 0

## Check Coefficients

# coef(cvfit, s = "lambda.1se")

coef.min = coef(cvfit, s = "lambda.min")

## riskscore

as.matrix(dat1[,c(-1,-2)]) %*% as.matrix(coef.min)

## plot

plot(cvfit)

plot(cvfit$glmnet.fit, xvar = "norm")

plot(cvfit$glmnet.fit, xvar = "lambda")

**Different Expressed Genes**

load('TCGA_XXX.RData')

tcga.data=log(dt+1)

load('TCGA_XXX.RData')

gtex.data=log(dt+1)

load('Clinical.RData')

gene.type=read.csv('GeneTag.txt',stringsAsFactors = F,row.names = 1,sep = '\t',check.names = F)

gene.type1=gene.type[which(gene.type$TYPE=='protein_coding'),]

nor.data=tcga.data[,grep('-11',colnames(tcga.data))]

comsamples=intersect(c(paste0(rownames(clinical),'-01'),paste0(rownames(clinical),'-03'),paste0(rownames(clinical),'-06')),colnames(tcga.data))

tum.data=tcga.data[,comsamples]

clin.cut=clinical[substr(comsamples,1,12),]

comgenes=intersect(intersect(rownames(tcga.data),rownames(gtex.data)),gene.type1$SYMBOL)

datas=cbind.data.frame(tum.data[comgenes,],nor.data[comgenes,],gtex.data[comgenes,])

gs.file=c(rep('Tumor',ncol(tum.data)),rep('Normal',ncol(nor.data)),rep('Normal',ncol(gtex.data)))

datas=datas[which(apply(datas,1,function(x){return(sum(x>0))})>0.5*ncol(datas)),]

limma_DEG=function(exp,group,ulab,dlab){

library(limma)

ind1=which(group==ulab)

ind2=which(group==dlab)

sml <- c(rep('G1',length(ind1)),rep('G0',length(ind2))) # set group names

eset=exp[,c(ind1,ind2)]

fl <- as.factor(sml)

design <- model.matrix(~fl+0)

colnames(design) <- levels(fl)

cont.matrix<-makeContrasts(contrasts='G1-G0',levels=design)

#print(head(eset))

fit<-lmFit (eset,design)

fit2 <- contrasts.fit(fit, cont.matrix)

fit2 <- eBayes(fit2)

#print(sml)

tT <- topTable(fit2, adjust="fdr", sort.by="B", number=nrow(eset))

return(list(Exp=eset,Group=group[c(ind1,ind2)],DEG=tT))

}

deg=limma_DEG(datas,gs.file,'Tumor','Normal')

deg.result=deg$DEG[which(abs(as.numeric(deg$DEG$logFC)) > 1&as.numeric(deg$DEG$FDR) < 0.05),]

up.genes=rownames(deg.result)[which(deg.result$logFC > 0)]

down.genes=rownames(deg.result)[which(deg.result$logFC < 0)]

Volcano=function(logfc,pvalue,symbol=NULL,cutFC=1,cutPvalue=0.05

,showText=NULL

,colors=c(mypal[2],'grey',mypal[1])

,xlim=NULL,ylim=NULL

,legend.pos='tl'

,ylab='-log10(FDR)',leg='',xlab='log2(FoldChange)'){

library(ggplot2)

pos=c(0,0)

if(is.null(legend.pos)){

pos='none'

}else if(legend.pos=='tr'){

pos=c(1,1)

}else if(legend.pos=='br'){

pos=c(1,0)

}else if(legend.pos=='tl'){

pos=c(0,1)

}else if(legend.pos=='bl'){

pos=c(0,0)

}else{

pos='right'

}

cange=rep('None',length(logfc))

cange[which(logfc>cutFC&pvalue<cutPvalue)]='Up-regulation'

cange[which(logfc< -cutFC&pvalue<cutPvalue)]='Down-regulation'

if(is.null(symbol)){

symbol=rep('',length(logfc))

showText=NULL

}

vo.input=data.frame(logFC=logfc,FDR=pvalue,change=cange,SYMBOL=symbol)

#print(head(vo.input))

p1 <- ggplot(data = vo.input,

aes(x = logFC,

y = -log10(FDR)))

if (ylab == 'FDR') {

p1=p1+geom_point(alpha=0.4, size=3.5, aes(color=change))+labs(x=bquote(~Log[2]~"(fold change)"), y=bquote(~-Log[10]~italic('FDR')), title="")

}else{

p1=p1+geom_point(alpha=0.4, size=3.5, aes(color=change))+labs(x=bquote(~Log[2]~"(fold change)"), y=bquote(~-Log[10]~italic('P-value')), title="")

}

p1=p1+scale_color_manual(values=colors,limits = c("Down-regulation",'None', "Up-regulation"),name=leg)+ scale_x_continuous(

breaks = c(-10, -5, -cutFC, 0, cutFC, 5, 10),

labels = c(-10, -5, -cutFC, 0, cutFC, 5, 10))

p1=p1+geom_vline(xintercept=c(-cutFC,cutFC),lty=4,col="black",lwd=0.8)

p1=p1+geom_hline(yintercept = -log10(cutPvalue),lty=4,col="black",lwd=0.8)

p1=p1+theme_bw()

p1=p1+theme(

axis.text.y=element_text(family="serif",face="plain"),

axis.title.y=element_text(family="serif",face="plain"),

legend.text=element_text(family="serif",face="plain", colour="black"

),

legend.title=element_text(family="serif",face="plain", colour="black"

),

legend.justification=pos, legend.position=pos

,legend.background = element_rect(fill = NA, colour = NA)

)

if(is.null(showText)|is.null(symbol)){

showText=c()

}

if(length(showText)>0){

for_label <-vo.input[match(intersect(showText,vo.input$SYMBOL),vo.input$SYMBOL),]

p1=p1+geom_point(size = 3, shape = 1, data = for_label)+ggrepel::geom_label_repel(

aes(label = SYMBOL),

data = for_label,

color="black"

)

}

if(!is.null(ylim)){

p1=p1+ylim(ylim)

}

if(!is.null(xlim)){

p1=p1+xlim(xlim)

}

p1=p1+theme(text=element_text(size=12,family="serif"))

return(p1)

}

gene_volcano=Volcano(as.numeric(deg$DEG$logFC),as.numeric(deg$DEG$FDR),cutFC = cut.fc,colors=c('red','grey','blue')

,cutPvalue = cut.p,symbol = rownames(deg$DEG),ylab = 'P-value',showText = symlist)

if (length(up.genes)>100){

genes1=rownames(deg.dif.result.final)[order(deg.dif.result.final$logFC,decreasing = T)][1:100]

}else{

genes1=up.genes

}

if (length(down.genes)>100){

genes2=rownames(deg.dif.result.final)[order(deg.dif.result.final$logFC)][1:100]

}else{

genes2=down.genes

}

indata=indata[c(genes1,genes2),]

indata=as.data.frame(remove_NA(indata))

annCol=as.data.frame(group)

rownames(annCol)=colnames(datas)

###################################

annColors <- list()

if (length(clinType)>1) {

annColors[["group"]] <- c("G1"="#EA6767","G2"="#70C17A")

}else{

annColors[["group"]] <- c("G1"="#EA6767","Normal"="#70C17A")

}

blank <- " "

add.label <- str_pad(rep(" ",nrow(indata)),

max(nchar(paste0(rownames(indata)))),

side = "right")

gene_map=pheatmap(mat = indata

,clustering_distance_rows = 'correlation'

,clustering_distance_cols = 'correlation'

,scale = "none"

,border_color = NA

,color = colorRampPalette(c(mypal1[2], "white", mypal1[1]))(100)

,cluster_cols = T

,cluster_rows = T

,show_rownames = T

,show_colnames = F

,annotation_col = annCol

#,annotation_row = annrow

,annotation_colors = annColors

,labels_row = paste(add.label,sep=blank))

fun_clusterProfiler=function(genes,minGSSize=10,maxGSSize = 500, pAdjustMethod = "BH",pvalueCutoff=0.05,fdrCutoff = 0.5,qvalueCutoff = 0.5){

library(org.Hs.eg.db)

#pathways=readMatrix('/opt/shengxin/app/pathway_gids.txt',header=F,row=F)

pathways=readMatrix('./pathway_gids.txt',header=F,row=F)

eid=AnnotationDbi::select(org.Hs.eg.db,keys = as.character(pathways[,2]),keytype = 'ENTREZID',columns = c('SYMBOL'))

pathways[,2]=eid$SYMBOL[match(pathways[,2],eid$ENTREZID)]

pathways=pathways[!is.na(pathways[,2]),]

#hg.tab=select(org.Hs.eg.db, keys=keys(org.Hs.eg.db,keytype = "ENTREZID"),columns=c('GO','GOALL')

# , keytype="ENTREZID")

pCut=pvalueCutoff

pvalueCutoff = fdrCutoff

#qvalueCutoff = 0.5

#print('Starting KEGG')

kegg=clusterProfiler::enricher(genes, pvalueCutoff = pvalueCutoff, pAdjustMethod = pAdjustMethod,

minGSSize = minGSSize, maxGSSize = maxGSSize, qvalueCutoff = qvalueCutoff

,TERM2GENE=data.frame(term =pathways[,1],gene=pathways[,2]),

TERM2NAME = data.frame(term =pathways[,1],name=pathways[,3]))

#print('Succ KEGG,Start GO_MF')

eid=AnnotationDbi::select(org.Hs.eg.db,keys = as.character(genes),keytype = 'SYMBOL',columns = c('ENTREZID'))

genes1=eid$ENTREZID

genes1=as.character(genes1[!is.na(genes)])

go.mf=clusterProfiler::enrichGO(genes1, org.Hs.eg.db, keyType = "ENTREZID", ont = "MF",

pvalueCutoff = pvalueCutoff, pAdjustMethod = pAdjustMethod,

qvalueCutoff = qvalueCutoff, minGSSize = minGSSize, maxGSSize = maxGSSize,

readable = T, pool = FALSE)

#print('Succ GO_MF,Start GO_CC')

go.cc=clusterProfiler::enrichGO(genes1, org.Hs.eg.db, keyType = "ENTREZID", ont = "CC",

pvalueCutoff = pvalueCutoff, pAdjustMethod = pAdjustMethod,

qvalueCutoff = qvalueCutoff, minGSSize = minGSSize, maxGSSize = maxGSSize,

readable = T, pool = FALSE)

#print('Succ GO_CC,Start GO_BP')

go.bp=clusterProfiler::enrichGO(genes1, org.Hs.eg.db, keyType = "ENTREZID", ont = "BP",

pvalueCutoff = pvalueCutoff, pAdjustMethod = pAdjustMethod,

qvalueCutoff = qvalueCutoff, minGSSize = minGSSize, maxGSSize = maxGSSize,

readable = T, pool = FALSE)

enrich_tab=rbind(cbind((kegg@result),DB=rep('pathway_KEGG',nrow(kegg@result)))

,cbind((go.bp@result),DB=rep('geneontology_Biological_Process',nrow(go.bp@result)))

,cbind((go.cc@result),DB=rep('geneontology_Cellular_Component',nrow(go.cc@result)))

,cbind((go.mf@result),DB=rep('geneontology_Molecular_Function',nrow(go.mf@result)))

)

enrich_tab=crbind2DataFrame(enrich_tab)

colnames(enrich_tab)[c(2,5,6,9)]=c('description','pValue','FDR','size')

enrich_tab$enrichmentRatio=unlist(lapply(strsplit(enrich_tab[,3],'/'), function(x){

x1=as.numeric(x)

return(x1[1]/x1[2])

}))

enrich_tab=enrich_tab[which(enrich_tab[,5]<pCut&enrich_tab[,6]<fdrCutoff&enrich_tab[,7]<pvalueCutoff),]

return(list(KEGG=kegg,GO_BP=go.bp,GO_CC=go.cc,GO_MF=go.mf,Enrich_tab=enrich_tab))

}

dotplot_batch=function(pathway_data,db,colors,top=20,FDR=T){

library(ggplot2)

#library(ggsci)

#db='pathway_KEGG'

#pathway_data=up.result$KEGG@result

if(nrow(pathway_data)>0){

if(nrow(pathway_data)>top){

pathway_data=pathway_data[order(pathway_data$p.adjust)[1:top],]

tl=paste0(db)

}else{

tl=paste0(db)

}

desc=pathway_data$Description

ndesc=c()

for(de in desc){

if(nchar(de)>50&length(grep(' ',de))>0){

de1=unlist(strsplit(de,' '))

d2=paste0(de1[(ceiling(length(de1)/2)+1):length(de1)],collapse = ' ')

if(nchar(d2)>50){

d2=paste0(substr(d2,0,47),'...')

}

de2=paste0(paste0(de1[1:ceiling(length(de1)/2)],collapse = ' '),'\n'

,d2)

ndesc=c(ndesc,de2)

}else{

ndesc=c(ndesc,de)

}

}

pathway_data$Description=ndesc

pathway_data$pvalue[pathway_data$pvalue<1e-16]=1e-16

pathway_data$p.adjust[pathway_data$p.adjust<1e-16]=1e-16

for (i in 1:nrow(pathway_data)) {

can11=as.data.frame(strsplit(pathway_data$GeneRatio[i],"/"),stringsAsFactors = FALSE)

pathway_data$size[i]=round(as.numeric(can11[1,])/as.numeric(can11[2,]),2)

}

pathway_data=pathway_data[order(pathway_data$size),]

bubble=ggplot(data = pathway_data, aes(x = size, y = Description))

bubble=bubble+xlab('Enrichment Ratio')

if(FDR){

bubble1=bubble+geom_point(aes(size = Count,color = -log10(p.adjust))) + scale_color_gradient(low = ggsci::pal_npg()(2)[2], high = ggsci::pal_npg()(2)[1])

bubble2=bubble+geom_point(aes(size = Count,color = -log10(p.adjust))) + scale_color_gradient(low = ggsci::pal_nejm()(2)[2], high = ggsci::pal_nejm()(2)[1])

bubble3=bubble+geom_point(aes(size = Count,color = -log10(p.adjust))) + scale_color_gradient(low = ggsci::pal_lancet()(2)[1], high = ggsci::pal_lancet()(2)[2])

bubble4=bubble+geom_point(aes(size = Count,color = -log10(p.adjust))) + scale_color_gradient(low = ggsci::pal_jama()(2)[1], high = ggsci::pal_jama()(2)[2])

bubble5=bubble+geom_point(aes(size = Count,color = -log10(p.adjust))) + scale_color_gradient(low = ggsci::pal_jco()(2)[1], high = ggsci::pal_jco()(2)[2])

}else{

bubble1=bubble+geom_point(aes(size = Count,color = -log10(pValue))) + scale_color_gradient(low = ggsci::pal_npg()(2)[2], high = ggsci::pal_npg()(2)[1])

bubble2=bubble+geom_point(aes(size = Count,color = -log10(pValue))) + scale_color_gradient(low = ggsci::pal_nejm()(2)[2], high = ggsci::pal_nejm()(2)[1])

bubble3=bubble+geom_point(aes(size = Count,color = -log10(pValue))) + scale_color_gradient(low = ggsci::pal_lancet()(2)[1], high = ggsci::pal_lancet()(2)[2])

bubble4=bubble+geom_point(aes(size = Count,color = -log10(pValue))) + scale_color_gradient(low = ggsci::pal_jama()(2)[1], high = ggsci::pal_jama()(2)[2])

bubble5=bubble+geom_point(aes(size = Count,color = -log10(pValue))) + scale_color_gradient(low = ggsci::pal_jco()(2)[1], high = ggsci::pal_jco()(2)[2])

}

if (colors=='npg') {

bubble=bubble1+ggsci::scale_fill_npg()+ggplot2::theme_bw()+theme(axis.title.y=element_blank()

,axis.text.y=element_text(family="serif",face="plain",size = 15)

,axis.text.x=element_text(family="serif",face="plain")

,plot.title = element_text(hjust = 0.5,family="serif",face="plain")

,axis.title.x=element_text(family="serif",face="plain")

,legend.title = element_text(family="serif",face="plain")

,legend.text = element_text(family="serif",face="plain"))

}else if(colors=='nejm'){

bubble=bubble2+ggsci::scale_fill_nejm()+ggplot2::theme_bw()+theme(axis.title.y=element_blank()

,axis.text.y=element_text(family="serif",face="plain",size = 15)

,axis.text.x=element_text(family="serif",face="plain")

,plot.title = element_text(hjust = 0.5,family="serif",face="plain")

,axis.title.x=element_text(family="serif",face="plain")

,legend.title = element_text(family="serif",face="plain")

,legend.text = element_text(family="serif",face="plain"))

}else if(colors=='lancet'){

bubble=bubble3+ggsci::scale_fill_lancet()+ggplot2::theme_bw()+theme(axis.title.y=element_blank()

,axis.text.y=element_text(family="serif",face="plain",size = 15)

,axis.text.x=element_text(family="serif",face="plain")

,plot.title = element_text(hjust = 0.5,family="serif",face="plain")

,axis.title.x=element_text(family="serif",face="plain")

,legend.title = element_text(family="serif",face="plain")

,legend.text = element_text(family="serif",face="plain"))

}else if(colors=='jama'){

bubble=bubble4+ggsci::scale_fill_jama()+ggplot2::theme_bw()+theme(axis.title.y=element_blank()

,axis.text.y=element_text(family="serif",face="plain",size = 15)

,axis.text.x=element_text(family="serif",face="plain")

,plot.title = element_text(hjust = 0.5,family="serif",face="plain")

,axis.title.x=element_text(family="serif",face="plain")

,legend.title = element_text(family="serif",face="plain")

,legend.text = element_text(family="serif",face="plain"))

}else if(colors=='jco'){

bubble=bubble5+ggsci::scale_fill_jco()+ggplot2::theme_bw()+theme(axis.title.y=element_blank()

,axis.text.y=element_text(family="serif",face="plain",size = 15)

,axis.text.x=element_text(family="serif",face="plain")

,plot.title = element_text(hjust = 0.5,family="serif",face="plain")

,axis.title.x=element_text(family="serif",face="plain")

,legend.title = element_text(family="serif",face="plain")

,legend.text = element_text(family="serif",face="plain"))

}

bubble=bubble+ggtitle(tl)

bubble=bubble+theme(text=element_text(size=12,family="serif"))

}

return(bubble)

}

up.result=fun_clusterProfiler(up.genes,pvalueCutoff = 0.5,fdrCutoff = 0.5,qvalueCutoff = 0.5)

down.result=fun_clusterProfiler(down.genes,pvalueCutoff = 0.5,fdrCutoff = 0.5,qvalueCutoff = 0.5)

a=dotplot_batch(up.result$KEGG@result,db = 'KEGG pathway (Up)',colors = 'nejm')

b=dotplot_batch(up.result$GO_BP@result,db = 'GO (Up)',colors = 'nejm')

c=dotplot_batch(down.result$KEGG@result,db = 'KEGG pathway (Down)',colors = 'nejm')

d=dotplot_batch(down.result$GO_BP@result,db = 'GO (Down)',colors = 'nejm')

fuji=ggpubr::ggarrange(a,b,c,d, ncol = 2, nrow = 2,labels = '',align = "hv")

Immune Score

load('Immu_score.RData')

im.score=Immu_score[colnames(data.ana),grep('EPI',colnames(Immu_score))]

cor_point=function(x,y,method='Pearson',top_col='#D55E00',right_col='#009E73'

,ylab='y expression',xlab='x expression',title=NULL

,marginal.type=c("histogram", "boxplot", "density", "violin", "densigram")[1]){

library(ggstatsplot)

dat=data.frame(X=x,Y=y)

tp='nonparametric'

if(method=='Pearson'|method=='pearson'){

tp='parametric'

}

g1=ggscatterstats(data = dat,

x = X,

y = Y

,type = tp

,xfill = top_col

,yfill = right_col

,xlab = xlab

,ylab=ylab

,marginal.type = marginal.type

,title = title)

return(g1)

}

#################################

plot.rs=list()

for (aaa in colnames(im.score)) {

plot.rs[[aaa]]=cor_point(x=as.numeric(risk),y=log2(as.numeric(im.score[,aaa])+1),top_col='red',right_col='blue'

,xlab=paste0('Riskscore')

,ylab=paste0('Log2 (',aaa,' expression)')

,marginal.type='density',method = 'spearman')

}

plot.rs

GS=as.data.frame(data.map$Groups)

rownames(GS)=rownames(im.score)

colnames(GS)='Groups'

plotdata <- t(scale(im.score,center = T))

plotdata[plotdata > 2] <- 2

plotdata[plotdata < -2] <- -2

blank <- " "

p.value <- pvalues

sigcode <- cut(as.numeric(p.value), c(0, 0.001, 0.01, 0.05, 0.1, 1),labels=c('***', '**', '*', '', ''))

sig.label <- as.character(sigcode)

p.label <- formatC(p.value,format = "e",digits = 2)

add.label <- str_pad(paste0(rownames(plotdata),sig.label),

max(nchar(paste0(rownames(plotdata),sig.label))),

side = "right")

plotdata=plotdata[,rownames(GS)]

#annCol=data.gs

#colnames(annCol) <- 'Group'

# colnames(annCol)[1] <- paste(str_pad(colnames(annCol)[1],

# max(nchar(paste0(rownames(plotdata),sig.label))),

# side = "right"),"P-value",sep = blank)

#names(annColors) <- colnames(annCol)[1]

aa1=pheatmap(mat = plotdata,

scale = "none",

annotation_col = GS,

color = colorRampPalette(c(mypal[2], "white", mypal[1]))(100),

#annotation_colors = annColors,

cluster_cols = F,

cluster_rows = T,

show_colnames = F,

show_rownames = T,

#annotation_legend = F,

labels_row = paste(add.label, p.label, sep=blank),

fontfamily = "mono")

#####################################################

load('XXX.RData')

cli.eac=read.csv('PMC6066282-TCGA-CDR-clinical.txt',stringsAsFactors = F,row.names = 1,check.names = F,sep = '\t')

comsample=intersect(paste0(rownames(cli.eac),'-01'),colnames(dt))

data.ana=log2(dt[,comsample]+1)

data.cli=cli.eac[substr(comsample,1,12),]

genes=c('CD274','CTLA4','HAVCR2','LAG3','PDCD1','PDCD1LG2','TIGIT','SIGLEC15')

dt.che=data.ana[genes,]

ttt0= dt.che %>%

rownames_to_column('Samples') %>%

pivot_longer(cols = 2:(ncol(dt.che)+1),names_to='Celltype',values_to='Values')

datas.final=as.data.frame(rbind(datas,datas.nor))

pvalues <- sapply(unique(datas.final$Type), function(x) {

if (length(unique(datas.final$Group))==2) {

res <- wilcox.test(Values ~ Group, data = datas.final[which(datas.final$Type=='CD274'),])

res$p.value

}else if (length(unique(datas.final$Group))>=3){

res <- kruskal.test(Values ~ Group, data = datas.final[which(datas.final$Type==x),])

res$p.value

}

})

pv <- data.frame(Type = unique(datas.final$Type), pvalue = pvalues)

pv$sigcode <- cut(as.numeric(pv$pvalue), c(0, 0.001, 0.01, 0.05, 0.1, 1),

labels=c('***', '**', '*', '', ''))

p1=ggboxplot(datas.final,x='Type',y='Values',color='Group',palette = "nejm",size = 0.5,shape=16,

add = "jitter",add.params=list(size=0.1),bxp.errorbar =T,width=0.55,outlier.shape=NA,ggtheme=theme_bw())

p1=p1+geom_text(aes(Type, y=max(datas.final$Values)*1.1,label=pv$sigcode),data=pv, inherit.aes=F,size=6) + xlab(NULL)+ylab('Immune checkpoint')

p1=p1+theme(axis.text.x=element_text(angle=45,hjust = 1,colour="black",family="serif",size = 10)

,axis.text.y=element_text(family="serif",face="plain",size = 10)

,axis.title.y=element_text(family="serif",face="plain",size = 10)

,panel.border = element_blank(),axis.line = element_line(colour = "black")

,legend.text=element_text(face="plain", family="serif", colour="black",size = 10)

,legend.title=element_text(face="plain", family="serif", colour="black",size = 10)

#,legend.justification=c(1,1), legend.position=c(1,1)

,legend.background = element_rect(fill = NA, colour = NA)

,panel.grid.major = element_blank()

,panel.grid.minor = element_blank())

if (length(unique(datas.final$Group))==2) {

fin.box=p1+labs(fill =paste0(" wilcox.test","\n\n"," * p < 0.05","\n\n","** p < 0.01","\n\n","Groups"))+theme(text=element_text(size=8,family="serif"))

}else if (length(unique(datas.final$Group))>=3){

fin.box=p1+labs(fill =paste0(" kruskal.test","\n\n"," * p < 0.05","\n\n","** p < 0.01","\n\n","Groups"))+theme(text=element_text(size=8,family="serif"))

}

library(ggsci)

mypal = pal_nejm(alpha = 0.7)(7)

plotKMCox=function(dat,genes,type,mypal){

colnames(dat)=c('time','status','groups')

dat=dat[which(dat[,1]!='NA'&dat[,2]!='NA'&dat[,3]!='NA'),]

gp=c('Low exp','High exp')

vls=1:length(gp)

gvls=vls[match(dat[,3],gp)]

dt=data.frame(data.frame(dat[,1],dat[,2],gvls))

aa=coxFun(dt)

fit<-survfit(Surv(time,status) ~ groups,data=dat)

# cox=coxph(Surv(time,status) ~ groups,data=dat)

# b=summary(cox)

hr=round(as.numeric(aa[2]),3)

lower.hr=round(as.numeric(aa[3]),3)

upper.hr=round(as.numeric(aa[4]),3)

p=round(as.numeric(aa[1]),3)

sdf<-survdiff(Surv(time,status) ~ groups,data=dat)

#p<-pchisq(sdf$chisq,length(sdf$n)-1,lower.tail=FALSE)

sf<-survfit(Surv(time,status) ~ groups,data=dat)

plot(sf, mark.time = TRUE,col=mypal,xlab=paste("survival years (",type," )"),ylab = "survival rate",main=genes,lwd=2,cex.axis=1.3,cex.lab=1.5,font=2)

legend('topright',paste0(gsub('groups=','',names(sf$strata)),' ( N = ',sdf$n,')'), col = mypal,

lty = c(1,1, 1, 1),lwd=c(1,1,1,1),merge = TRUE,cex = 1.2)

text(x=max(fit$time)/2,y=0.1,paste("log-rank P=",signif(p, digits = 3),"\n","HR=",signif(hr,3),"(","95%CI,",signif(lower.hr,3),"-",signif(upper.hr,3),")"),

bty="n",font=2)

return(p)

}

data.fin=data.frame(time=times,status=status,t(data.analys))

data.fin=data.fin[which(data.fin$time!='NA'&data.fin$status!='NA'),]

plotKMCox(data.frame(data.fin$time,data.fin$status,label),ii,timeType,mypal)

###############

sigcox=as.data.frame(tra.cox[genes,])

dat.test=data.frame(Uni_cox=rownames(sigcox),Pvalue=ifelse(round(sigcox$p.value,3)==0,'<0.0001',round(sigcox$p.value,3))

,round(sigcox$HR,3),round(sigcox$`Low 95%CI`,3),round(sigcox$`High 95%CI`,3))

pdf('Figure31.pdf',width = 8,height = 6)

forestplot_v1(dat.test,xlog = T,colgap = 8,lineheight = 10,xlab = 'Hazard Ratio',box_col=mypal[1],summary_col='black',graph.pos=4)

dev.off()

forestplot_v1=function(dat,show_95CI=T,zero = 1,boxsize = 0.4,lineheight =5,colgap =2,lwd.zero=2,lwd.ci=2

,box_col='#458B00',summary_col="#8B008B",lines_col='black',zero_col='#7AC5CD'

,xlab='HR',lwd.xaxis=2,lty.ci = "solid",graph.pos = 2,xlim=NULL,xlog=F){

nc=ncol(dat)

nr=nrow(dat)

library(forestplot)

col=fpColors(box=box_col,summary=summary_col,lines = lines_col,zero = zero_col)

if(nc>3){

hr=as.numeric(dat[,nc-2])

lower=as.numeric(dat[,nc-1])

upper=as.numeric(dat[,nc])

if(is.null(xlim)){

xlim=c(min(lower,na.rm = T),max(upper,na.rm = T))

}

if(is.infinite(max(xlim))){

xlim=c(xlim[1],5)

}

if(is.infinite(min(xlim))){

xlim=c(0,xlim[2])

}

if(min(xlim)<=0){

xlog=F

}

smary=rep(F,length(hr))

nind=which(is.na(lower)|is.na(upper)|is.na(hr))

smary[nind]=T

labeltext=as.matrix(dat[,1:(nc-3)])

if(show_95CI){

adt=paste0(round(hr,5),'(',round(lower,5),',',round(upper,5),')')

adt[nind]=''

labeltext=cbind(labeltext,adt)

colnames(labeltext)=c(colnames(labeltext)[1:(ncol(labeltext)-1)],'Hazard Ratio(95% CI)')

}

if(graph.pos>ncol(labeltext)+1){

labeltext=ncol(labeltext)+1

}else if(graph.pos<2){

graph.pos=2

}

hz_list=list('2'=gpar(lty=1,col=summary_col),

'3'=gpar(lty=1,col=summary_col)

)

names(hz_list)=c(2,nrow(labeltext)+2)

p=forestplot(labeltext = rbind(colnames(labeltext),labeltext),

hrzl_lines = hz_list,

mean = c(NA,hr),

lower =c(NA,lower),

upper = c(NA,upper),

is.summary=c(T,smary),

zero = zero,

fn.ci_norm="fpDrawDiamondCI",

boxsize = boxsize,

lineheight = unit(lineheight,'mm'),

colgap = unit(colgap,'mm'),

lwd.zero = lwd.zero,

lwd.ci = lwd.ci,

col=col,

xlab=xlab,

lwd.xaxis=lwd.xaxis,

lty.ci = lty.ci,

clip = xlim,

#xlog=xlog,

mar=unit(rep(1.25, times = 4), "cm"),

txt_gp = fpTxtGp(ticks = gpar(cex = 0.8), xlab = gpar(cex = 1), cex = 0.8),

graph.pos = graph.pos,

new_page = F

)

return(p)

}else{

return(mg_getplot_bank('data must be greater than 3 column'))

}

}

##################

mg_limma_DEG=function(exp,group,ulab,dlab){

library(limma)

ind1=which(group==ulab)

ind2=which(group==dlab)

sml <- c(rep('G1',length(ind1)),rep('G0',length(ind2))) # set group names

eset=exp[,c(ind1,ind2)]

fl <- as.factor(sml)

design <- model.matrix(~fl+0)

colnames(design) <- levels(fl)

cont.matrix<-makeContrasts(contrasts='G1-G0',levels=design)

#print(head(eset))

fit<-lmFit (eset,design)

fit2 <- contrasts.fit(fit, cont.matrix)

fit2 <- eBayes(fit2)

#print(sml)

tT <- topTable(fit2, adjust="fdr", sort.by="B", number=nrow(eset))

return(list(Exp=eset,Group=group[c(ind1,ind2)],DEG=tT))

}

deg=mg_limma_DEG(datas,group,'G1','Normal')

up.genes=rownames(deg)[which(deg.dif.result.final$logFC > 1&as.numeric(deg.dif.result$adj.P.Val) < 0.05)]

down.genes=rownames(deg)[which(deg.dif.result.final$logFC < -1&as.numeric(deg.dif.result$adj.P.Val) < 0.05)]

fun_clusterProfiler=function(genes,minGSSize=10,maxGSSize = 500, pAdjustMethod = "BH",pvalueCutoff=0.05,fdrCutoff = 0.5,qvalueCutoff = 0.5){

library(org.Hs.eg.db)

#pathways=readMatrix('/opt/shengxin/app/pathway_gids.txt',header=F,row=F)

pathways=readMatrix('./pathway_gids.txt',header=F,row=F)

eid=AnnotationDbi::select(org.Hs.eg.db,keys = as.character(pathways[,2]),keytype = 'ENTREZID',columns = c('SYMBOL'))

pathways[,2]=eid$SYMBOL[match(pathways[,2],eid$ENTREZID)]

pathways=pathways[!is.na(pathways[,2]),]

#hg.tab=select(org.Hs.eg.db, keys=keys(org.Hs.eg.db,keytype = "ENTREZID"),columns=c('GO','GOALL')

# , keytype="ENTREZID")

pCut=pvalueCutoff

pvalueCutoff = fdrCutoff

#qvalueCutoff = 0.5

#print('Starting KEGG')

kegg=clusterProfiler::enricher(genes, pvalueCutoff = pvalueCutoff, pAdjustMethod = pAdjustMethod,

minGSSize = minGSSize, maxGSSize = maxGSSize, qvalueCutoff = qvalueCutoff

,TERM2GENE=data.frame(term =pathways[,1],gene=pathways[,2]),

TERM2NAME = data.frame(term =pathways[,1],name=pathways[,3]))

#print('Succ KEGG,Start GO_MF')

eid=AnnotationDbi::select(org.Hs.eg.db,keys = as.character(genes),keytype = 'SYMBOL',columns = c('ENTREZID'))

genes1=eid$ENTREZID

genes1=as.character(genes1[!is.na(genes)])

go.mf=clusterProfiler::enrichGO(genes1, org.Hs.eg.db, keyType = "ENTREZID", ont = "MF",

pvalueCutoff = pvalueCutoff, pAdjustMethod = pAdjustMethod,

qvalueCutoff = qvalueCutoff, minGSSize = minGSSize, maxGSSize = maxGSSize,

readable = T, pool = FALSE)

#print('Succ GO_MF,Start GO_CC')

go.cc=clusterProfiler::enrichGO(genes1, org.Hs.eg.db, keyType = "ENTREZID", ont = "CC",

pvalueCutoff = pvalueCutoff, pAdjustMethod = pAdjustMethod,

qvalueCutoff = qvalueCutoff, minGSSize = minGSSize, maxGSSize = maxGSSize,

readable = T, pool = FALSE)

#print('Succ GO_CC,Start GO_BP')

go.bp=clusterProfiler::enrichGO(genes1, org.Hs.eg.db, keyType = "ENTREZID", ont = "BP",

pvalueCutoff = pvalueCutoff, pAdjustMethod = pAdjustMethod,

qvalueCutoff = qvalueCutoff, minGSSize = minGSSize, maxGSSize = maxGSSize,

readable = T, pool = FALSE)

enrich_tab=rbind(cbind((kegg@result),DB=rep('pathway_KEGG',nrow(kegg@result)))

,cbind((go.bp@result),DB=rep('geneontology_Biological_Process',nrow(go.bp@result)))

,cbind((go.cc@result),DB=rep('geneontology_Cellular_Component',nrow(go.cc@result)))

,cbind((go.mf@result),DB=rep('geneontology_Molecular_Function',nrow(go.mf@result)))

)

enrich_tab=crbind2DataFrame(enrich_tab)

colnames(enrich_tab)[c(2,5,6,9)]=c('description','pValue','FDR','size')

enrich_tab$enrichmentRatio=unlist(lapply(strsplit(enrich_tab[,3],'/'), function(x){

x1=as.numeric(x)

return(x1[1]/x1[2])

}))

enrich_tab=enrich_tab[which(enrich_tab[,5]<pCut&enrich_tab[,6]<fdrCutoff&enrich_tab[,7]<pvalueCutoff),]

return(list(KEGG=kegg,GO_BP=go.bp,GO_CC=go.cc,GO_MF=go.mf,Enrich_tab=enrich_tab))

}

up.result=fun_clusterProfiler(up.genes,pvalueCutoff = 0.5,fdrCutoff = 0.5,qvalueCutoff = 0.5)

down.result=fun_clusterProfiler(down.genes,pvalueCutoff = 0.5,fdrCutoff = 0.5,qvalueCutoff = 0.5)

##################

colType1 = 'nejm'

p1=ggboxplot(datas,x='Type',y='values',color='Type',palette = "nejm",shape=16,size = 0.5,

add = "jitter",add.params=list(size=0.25),bxp.errorbar =T,width=0.5,outlier.shape=NA,ggtheme=theme_bw())

p1=p1+theme(axis.text.x=element_text(hjust = 0.5,colour="black",family="serif",size = 12)

,axis.text.y=element_text(family="serif",face="plain",size = 10)

,axis.title.y=element_text(family="serif",face="plain",size = 12)

,panel.border = element_blank(),axis.line = element_line(colour = "black")

,legend.text=element_text(face="plain", family="serif", colour="black",size = 12)

,legend.title=element_text(face="plain", family="serif", colour="black",size = 12)

#,legend.justification=c(1,1), legend.position=c(1,1)

,legend.background = element_rect(fill = NA, colour = NA)

,panel.grid.major = element_blank()

,panel.grid.minor = element_blank()

)+ylab(paste0(genes,' expression'))+xlab('')

aa1=p1+ggtitle(til)+theme(text=element_text(size=12,family="serif"))
